# SG-APSIC1102: Antimicrobial resistance and related factors in an intensive care unit—A study at Hue Central Hospital

**DOI:** 10.1017/ash.2023.83

**Published:** 2023-03-16

**Authors:** Tan Dang, Mi Ho Thihoa, Vo Dai Quyen, Tran Chi Thanh, Mai Van Tuan, Nguyen Thithanh Nhan, Le Diem Phuoc, Ha Thithuan, Hoang Thiquynh Nhi, Tran Thanh Thuy, Nguyen Van Thanh Toan, Hoang Thilan Huong

**Affiliations:** Hue Central Hospital, Hue City, Vietnam; Hue Central Hospital, Hue City, Vietnam; Hue Central Hospital, Hue City, Vietnam; Hue Central Hospital, Hue City, Vietnam; Hue Central Hospital, Hue City, Vietnam; Hue Central Hospital, Hue City, Vietnam; Hue Central Hospital, Hue City, Vietnam; Hue Central Hospital, Hue City, Vietnam; Hue Central Hospital, Hue City, Vietnam; Hue Central Hospital, Hue City, Vietnam; Hue Central Hospital, Hue City, Vietnam; Hue Central Hospital, Hue City, Vietnam

## Abstract

**Objectives:** Antimicrobial resistance (AMR) has emerged as a major concern in Vietnam, mainly due to the inappropriate use of antibiotics. Appropriate antibiotic management enables us to minimize the likelihood of antibiotic resistance and the spread of resistant bacteria. We evaluated vancomycin and colistin resistance and related factors in the intensive care unit (ICU) of Hue Central Hospital, a national hospital in central Vietnam. **Methods:** Using a cross-sectional descriptive study, we enrolled 362 patients who were prescribed antibiotics and were admitted to the ICU in 2019. Pathogens isolated from 473 routine clinical samples were subjected to antimicrobial susceptibility testing following the recommendations in the *Clinical & Laboratory Standards Institute M100, 28^th^* *Edition.* Colistin testing was performed using the broth microdilution method. Statistical significance was determined using the Fisher exact test. **Results:** The most commonly identified microorganisms were *Acinetobacter baumannii* (31.5%), *Klebsiella pneumoniae* (31.2%), *Pseudomonas aeruginosa* (12%), and *Staphylococcus aureus* (8.9%). All isolates of *A. baumannii*, *K. pneumoniae*, and *P. aeruginosa* tested with colistin were nonresistant. Moreover, >65% of *A. baumannii* isolates were resistant to all antibiotics except colistin. *S. aureus* had the highest resistance rate to erythromycin (80.6%), but no vancomycin-resistant isolates were identified. Factors associated with resistance to at least 1 antibiotic tested included length of stay (OR, 5.32; 95% CI, 1.47–19.17; *P* = .017), duration of antibiotics therapy (OR, 5.25; 95% CI, 1.46–18.95; *P* = .017), and the use of tracheal intubation and ventilator (OR, 3.08; 95% CI, 1.09–8.72; *P* = .038). **Conclusions:** These data indicated that although the vancomycin and colistin resistance rate is low, patients with longer length of stay, longer time on antibiotics, and invasive ventilation were at higher risk of AMR infection. Decreasing device use and strong antibiotic stewardship program at the hospital would help to reduce AMR infections.

